# A novel, wearable, electronic visual aid to assist those with reduced peripheral vision

**DOI:** 10.1371/journal.pone.0223755

**Published:** 2019-10-15

**Authors:** Ffion E. Brown, Janice Sutton, Ho M. Yuen, Dylan Green, Spencer Van Dorn, Terry Braun, Angela J. Cree, Stephen R. Russell, Andrew J. Lotery

**Affiliations:** 1 Clinical and Experimental Sciences, Faculty of Medicine, University of Southampton, University Hospital Southampton, Tremona Road, Southampton, England, United Kingdom; 2 Primary Care and Population Sciences, Faculty of Medicine, University of Southampton, University Hospital Southampton, Tremona Road, Southampton, England, United Kingdom; 3 Department of Ophthalmology and Visual Sciences, Carver College of Medicine, University of Iowa, Iowa City, IA, United States of America; 4 Southampton Eye Unit, University Hospital Southampton NHS Foundation Trust, University Hospital Southampton, Southampton, England, United Kingdom; Universita degli Studi di Firenze, ITALY

## Abstract

**Purpose:**

To determine whether visual-tactile sensory substitution utilizing the Low-vision Enhancement Optoelectronic (LEO) Belt prototype is suitable as a new visual aid for those with reduced peripheral vision by assessing mobility performance and user opinions.

**Methods:**

Sighted subjects (n = 20) and subjects with retinitis pigmentosa (RP) (n = 6) were recruited. The LEO Belt was evaluated on two cohorts: normally sighted subjects wearing goggles to artificially reduce peripheral vision to simulate stages of RP progression, and subjects with advanced visual field limitation from RP. Mobility speed and accuracy was assessed using simple mazes, with and without the LEO Belt, to determine its usefulness across disease severities and lighting conditions.

**Results:**

Sighted subjects wearing most narrowed field goggles simulating most advanced RP had increased mobility accuracy (44% mean reduction in errors, p = 0.014) and self-reported confidence (77% mean increase, p = 0.004) when using the LEO Belt. Additionally, use of LEO doubled mobility accuracy for RP subjects with remaining visual fields between 10° and 20°. Further, in dim lighting, confidence scores for this group also doubled. By patient reported outcomes, subjects largely deemed the device comfortable (100%), easy to use (92.3%) and thought it had potential future benefit as a visual aid (96.2%). However, regardless of severity of vision loss or simulated vision loss, all subjects were slower to complete the mazes using the device.

**Conclusions:**

The LEO Belt improves mobility accuracy and therefore confidence in those with severely restricted peripheral vision. The LEO Belt’s positive user feedback suggests it has potential to become the next generation of visual aid for visually impaired individuals. Given the novelty of this approach, we expect navigation speeds may improve with experience.

## Introduction

In 2010, inherited retinal diseases (IRDs) became the most common registerable cause of visual loss in England and Wales amongst the working age population (18–64).[1] Gene modifying or cellular replacement interventions are being developed for an increasing variety of IRDs, with one being approved by the FDA.[2, 3] Virtually all these therapeutic efforts however are directed at preserving or improving primarily central visual function, although most IRDs also result in profound and typically progressive peripheral visual loss. Additional approaches will be needed to provide or augment peripheral vision.

**Fig 1:**
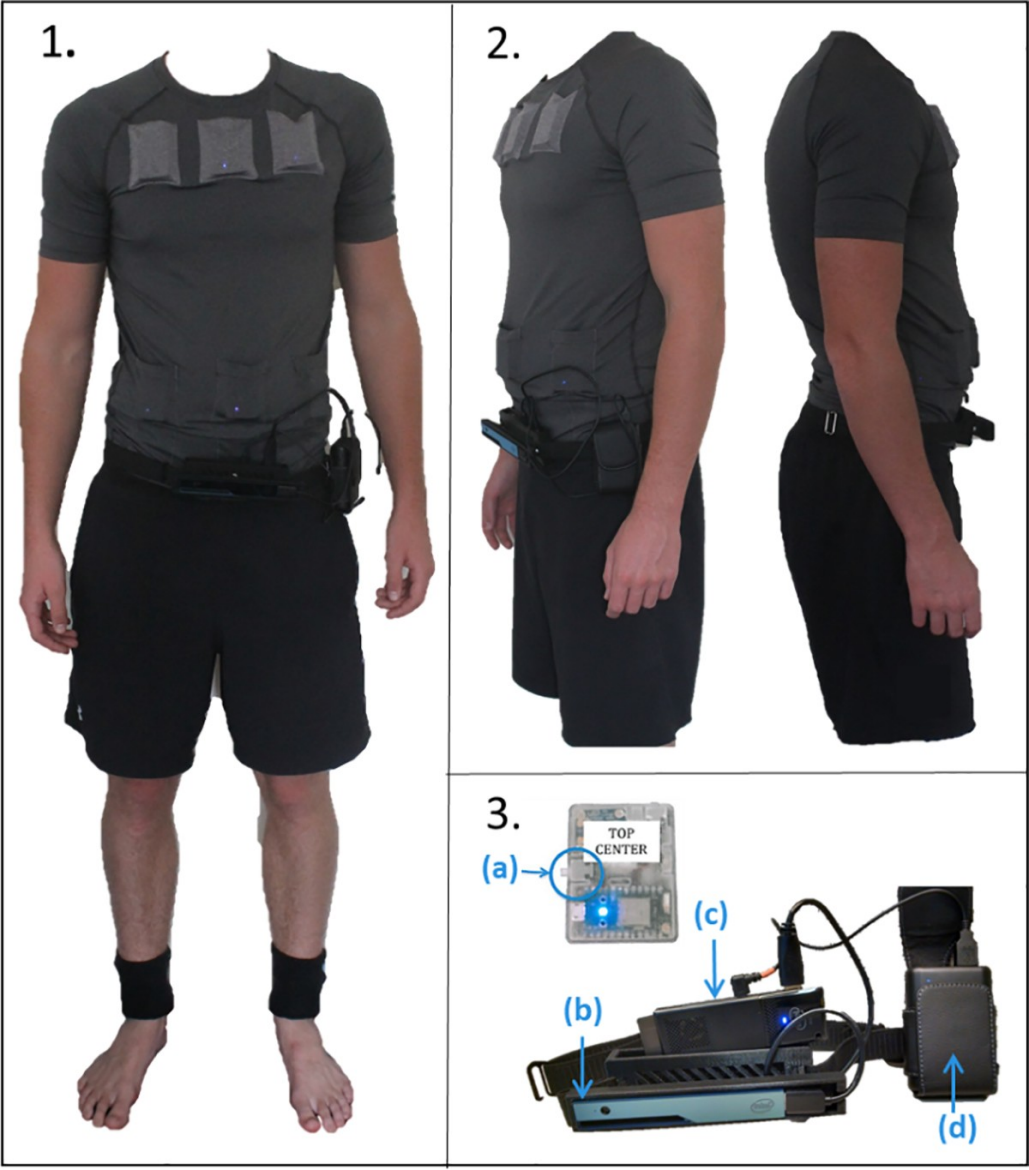
Components of the LEO Belt. Images of the LEO Belt showing the location of the eight vibration transducers, including two mounted on the ankles, which vibrate at increasing intensity to signify distance to an object between 0.5-2m (1). The 3D camera is belt-mounted with portable battery (1 and 2, profile view). The top centre transducer also contains the vibration on/off button (3a). The belt is formed of an Intel RealSense 3D camera (3b), Intel Compute Stick (3c) and portable battery (3d).

Toward this end, a number of wearable Electronic Travel Aids (ETAs) have been investigated, ranging from clip-on, auditory-feedback proximity detectors to an FDA-approved camera-based device utilising tongue stimulation as the haptic feedback method (Brainport V100, Madison, WI, USA).[4] However, no device thus far has been widely accepted by the blind community.[5] In 2015, Intel Corporation announced and provided open source software for a novel wearable device to assist retinitis pigmentosa (RP) patients with ambulation by enhancing their peripheral visual detection, a device they termed the Intel RealSense Awareness Wearable (IRSAW).[6] In collaboration with original designers, we modified the ergonomics and fabricated a small number of devices termed the Low-vision Enhancement Optoelectronic (LEO) Belt. The LEO Belt consists of a belt-mounted 3D depth-sensing camera (R200 Intel RealSense Camera, Intel Corp, Mountain View, CA), portable computer (Intel ComputeStick) and vibration transducers (six across the undershirt and one per ankle) (Fig 1).[7] The camera detects objects and distance in its field of view. The worn vibration transducers, connected to the camera and computer system via Wi-Fi, vibrate relative to spatial orientation of an object. For example, an object in the centre-right side of camera’s field of view would correspond to the right, middle vibration transducer. Distance is represented by vibration frequency, with a higher frequency corresponding to shorter distance.

The LEO Belt is unusual as it is designed specifically for those with reduced peripheral vision,[7] whereas most ETAs are designed for general visual impairment.[8] Reduced peripheral vision can result from numerous ocular diseases including glaucoma and RP, the latter affecting approximately 1:4000 worldwide.[9, 10] RP typically presents at ages between 20–30 years with nyctalopia, due to damaged rod photoreceptors, and bilateral ring scotomas upon visual field (VF) examination.[11] In many cases, progressive reduction in peripheral vision causes severe visual impairment by age 40–50, cone photoreceptors are subsequently affected and central vision lost.[12–14] RP occurs as a syndrome in 20–30% of patients, the most common being Usher syndrome.[14, 15] Prognosis for RP is poor as current treatments are limited to slowing disease progression and managing the daily consequences of reduced vision.[15]

The impaired mobility due to RP is the consequential limitation that most affects quality of life (QOL).[16] Navigation is slower and less accurate compared to controls; outcomes that are exaggerated in reduced lighting and partially reversed by using mobility aids.[17, 18] To objectively determine how reduced peripheral vision affects functional ability, standardised tools such as ‘Assessment of Disability Related to Vision’ (ADREV) have been developed.[19] If mobility is being assessed, the ADREV obstacle course can be used in isolation. ADREV error score divided by time (ADREV error/time) is the most predictive measurement of visual disease, as opposed to either time taken or number of errors alone.[20]

For those with impaired vision, other senses may be stimulated to substitute for visual input, typically utilising haptic or auditory approaches.[21] Traditional visual aids, such as a white cane, employ direct tactile information to assist navigation.[22] However, use of white canes and guide dogs are relatively low (between 17–50% and 2%, respectively) possibly due to social stigma.[23–25] Those not using aids often rely on relatives or friends, reducing their independence.[26] Improvements in technology has led to the development of a range of ETAs, but these are also only regularly used by 2% of visually impaired people.[5] As consensus has not been reached, current ETAs use a variety of input and output methods to translate visual information. Input sensors used thus far include ultrasound, infrared, cameras and lasers, each possessing limitations (S1 Table),[27] whereas the LEO Belt uses a 3D camera.[7] The LEO Belt conveys information via haptic stimulation as, unlike the auditory system, “the skin is rarely ever busy”.[28] Delivery of information is faster; however, interpretation is slower due to limits on our ability to notice haptic stimuli. The LEO Belt delivers vibrations to the torso, as areas that are more sensitive are impractical or may be unavailable at all times (such as the tongue or hands).[29–31] The limited tactile acuity (2-point discrimination) of the torso may restrict the amount of information that is perceived successfully.[22] However, this may be beneficial as simplicity minimises sensory and cognitive load, enabling prioritisation of immediately important information to avoid collisions.[8]

The LEO Belt’s novel 3D camera, discrete appearance and simple design specific to the functional requirements of those with reduced peripheral vision, suggest it has potential to become the new generation of visual aid. This study is the first to investigate the LEO Belt and utilises subjects with simulated VF loss and subjects with advanced organic reduced peripheral vision to assess mobility performance and user opinions. Furthermore, the cohort to benefit most from the device will be determined by investigating relationships to both disease severity and lighting conditions.

## Methods

### Experimental protocol

Twenty-six subjects were recruited; 20 sighted and 6 visually impaired subjects with diagnoses of RP, best corrected visual acuity (BCVA) <6/18 and residual central VF <20° bilaterally. Informed written consent was obtained according to protocols approved by Yorkshire & The Humber—Leeds West Research Ethics Committee (IRAS ID: 229062) and the University of Southampton Ethics and Research and Governance Online committee (ERGO: 31887 and 30421). The individual photographed wearing the device in this manuscript (Fig 1) has given written informed consent (as outlined in PLOS consent form) to publish these images. This study was carried out according to the tenets of the Declaration of Helsinki.

**Fig 2:**
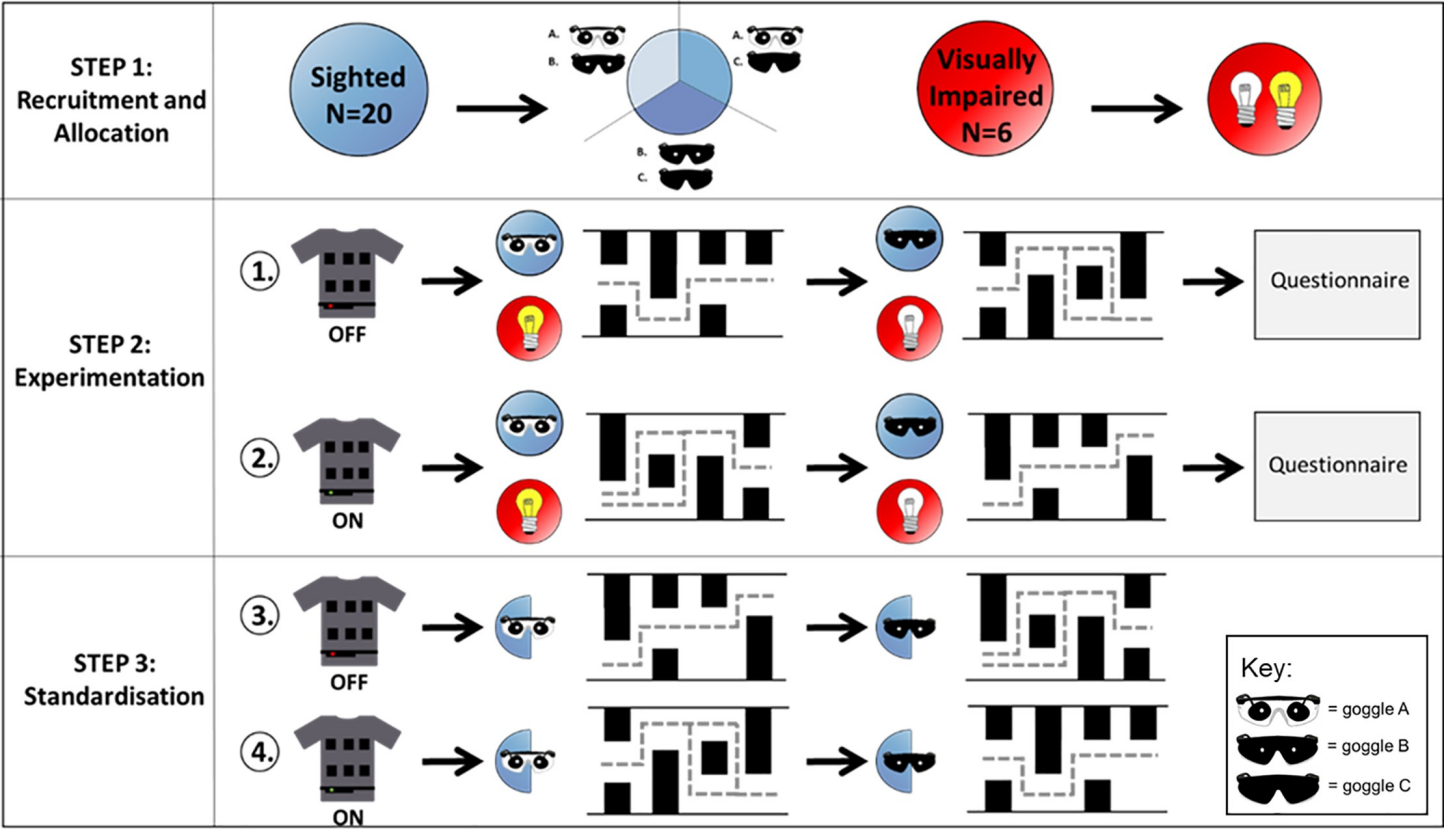
Experimental protocol. Visual representation demonstrating the three steps of the experimental protocol. Sighted subjects (blue) were allocated goggles (A-C) to reduce their vision before completing the mazes. Visually impaired subjects (red) completed the mazes in both bright and dim light. Half of the sighted subjects (blue semicircle) repeated the mazes with the variants in a different order.

The LEO Belt was introduced and each subject practiced for a maximum of ten minutes before mobility testing. All subjects then walked through four variations (A to D) of a simple maze in a random order; the 1^st^ and 2^nd^ mazes were without using the LEO Belt followed by the 3^rd^ and 4^th^ mazes with LEO Belt (Fig 2). Sighted subjects were given two of three pairs of goggles in sequence to simulate reduced VFs, one pair worn for the 1^st^ and 3^rd^ mazes and the other for the 2^nd^ and 4^th^. The goggles each represented a different stage of RP disease severity; goggle A represented characteristic ring scotomas with no vision between 10–50°, goggle B represented severe reduced peripheral vision with only central 10° vision remaining and goggle C represented nil-perception of light (NPL) (S2 Table). Half of the sighted subject cohort repeated the four maze attempts but with the maze variants in a different order. This was done to ensure that no variant significantly differed in its’ difficulty. Visually impaired subjects were permitted to use their normal visual aid if required during testing, including as an adjunct to the LEO Belt. They tested the device at two lighting levels, bright and dim lighting to determine possible impact of nyctalopia. Luminance was measured using a mobile application (Lux Meter) with illuminance levels >200 lux being defined as bright lighting and <10 lux for dim lighting. Finally, all subjects completed two short questionnaires, one before and one after using the LEO Belt (S1 Fig).

### Maze design and assessment

**Fig 3:**
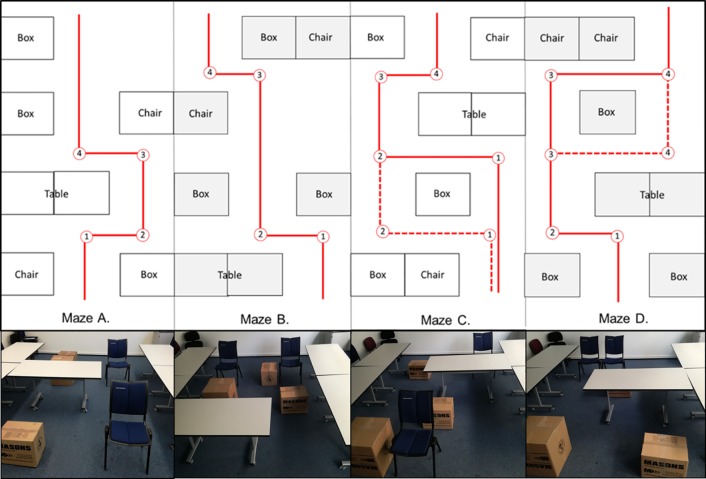
Maze variants. Diagrammatic and photographic images of the four maze variants used (A to D), showing that each variant contains seven objects and four directional changes.

Each maze variant contained four directional changes and used the same seven objects: three boxes, two chairs and two small desk tables (equivalent to one large desk table) (Fig 3).The LEO Belt was worn throughout the experiment and activated to test the LEO Belt, and deactivated when not under test. Maze attempts were recorded using a video camera. Measured outcomes were the time taken to complete each maze and the number of errors made. An error was defined as an object hit or near-hit, the latter occurring when the researcher intervened vocally to prevent collision. Each maze attempt was scored as one point per hit or near-hit object, with a maximum of 7 errors, reflecting the validated ADREV error scoring system.[20] To produce a combined time and error score, the error score was first inverted so that 7 represented a perfect score and 0 the maximum number of errors. This was then converted to a 1–8 scale to remove a 0 numerator. This score was divided by time taken in seconds to produce a combined metric, called ADREV error/time, whereby smaller values represent poorer performance.[20]

### Data analysis

Statistical analysis was carried out using IMB SPSS Statistics version 24 and GraphPad Prism version 7 software. Shapiro-Wilk test was applied to determine data distribution. Univariate Analysis of Variance (ANOVA) was conducted to verify the maze model. Parametric and non-parametric variables, measuring time, number of errors and confidence scores, were analysed using a paired samples t-test or Wilcoxon singed ranks test respectively. Correlations were analysed using Pearson correlation coefficient or Spearman’s rank correlation according to data distribution pattern. Results with p<0.05 were considered statistically significant.

## Results

Subjects had a mean age of 37.6 (range 22 to 83); 30.4 years for sighted subjects and 61.5 years for visually impaired subjects. Gender distribution (12 male and 14 female) was balanced. Among the subjects with RP, three had Usher syndrome, the other three had genetically uncharacterised RP. Subjects with severely limited VA were unable to complete VF testing as they could only see hand movements (HM) or less (S3 Table for VF results). For analysis, subjects were divided into two groups; those with sufficient vision to have measurable VFs and those with unmeasurable VFs (Table 1). Only one subject with unmeasurable VFs reported experiencing nyctalopia. Three visually impaired subjects used a white cane during testing, one used a guide dog and two used no aid. Of those using visual aids, only one subject tested the LEO Belt without it as an adjunct.

**Table 1:** Visually impaired subject characteristics.

ID	Gender	Age	Diagnosis	BCVA OS	BCVA OD	VF OS	VF OD	Nyctalopia	Reliance on Visual Aid
V01	Female	78	Usher syndrome	NPL	HM	n/a	n/a	No	Yes
V02	Male	50	Bilateral RP	HM	HM	n/a	n/a	Yes	Yes
V03	Male	59	Bilateral RP	PL/NPL	PL	n/a	n/a	No	Yes
V04	Female	52	Usher syndrome	CF	6/18	20°	20°	Yes	No
V05	Female	83	Bilateral RP	HM	6/18	10°	10°	Yes	Yes
V06	Male	47	Usher syndrome	6/30	6/30	10°	10°	Yes	No

Key: RP = retinitis pigmentosa, NPL = nil-preception of light, PL = perception of light

CF = counting fingers, HM = hand movements

BCVA = best corrected visual acuity, VF = visual fields

OS = oculus sinister (left eye), OD = oculus dexter (right eye)

Demographic, diagnostic and background data from visually impaired subjects. Subjects have been dichotomised into two groups, those with unmeasurable visual fields (grey) and measurable visual fields (white).

**Fig 4:**
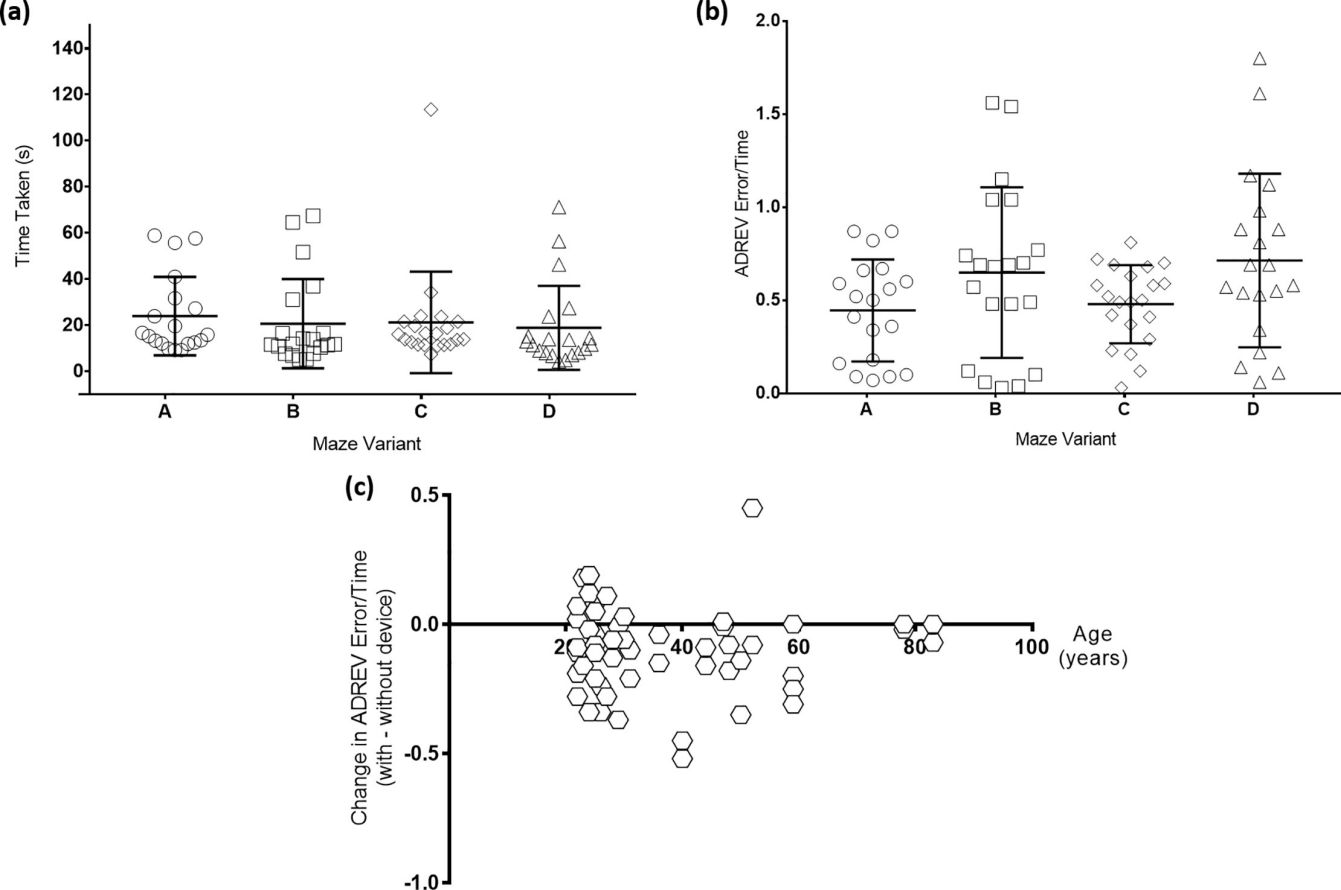
Analysis of confounding variables. Repeated maze attempts from sighted subjects revealed that maze variant (A to D) used had no significant effect on time taken to complete each maze, measured in seconds, (p = 0.380) (a) or ADREV error/time scores (p = 0.182) (b). Age of all subjects, in years, was found to not be statistically correlated with change in ADREV error/time score (p = 0.9187) (c).

Among sighted subjects, ANOVA testing found that using goggles (A to C) significantly increased the completion time (p<0.001), mirroring the effect of increasing disease severity in RP. Results from the sighted subject cohort who repeated the four mazes twice, were analysed to determine the effect of the order of the mazes on time taken. This revealed that there was no statistical difference in time taken between the 1^st^ and 2^nd^ maze attempts (when the LEO Belt was electronically inactivated) or the 3^rd^ and 4^th^ maze attempts (when the LEO Belt was electronically activated) (p = 1.000 for both). The maze variant used (A to D) also did not statistically alter time taken (p = 0.380) (Fig 4A) or ADREV errors/time scores (p = 0.182) (Fig 4B). This result was obtained by including all maze attempts, with and without the LEO Belt. Despite different age characteristics of the sighted and visually impaired cohorts, there was no significant correlation between age and change in ADREV error/time scores (p = 0.9187) (Fig 4C).

**Fig 5:**
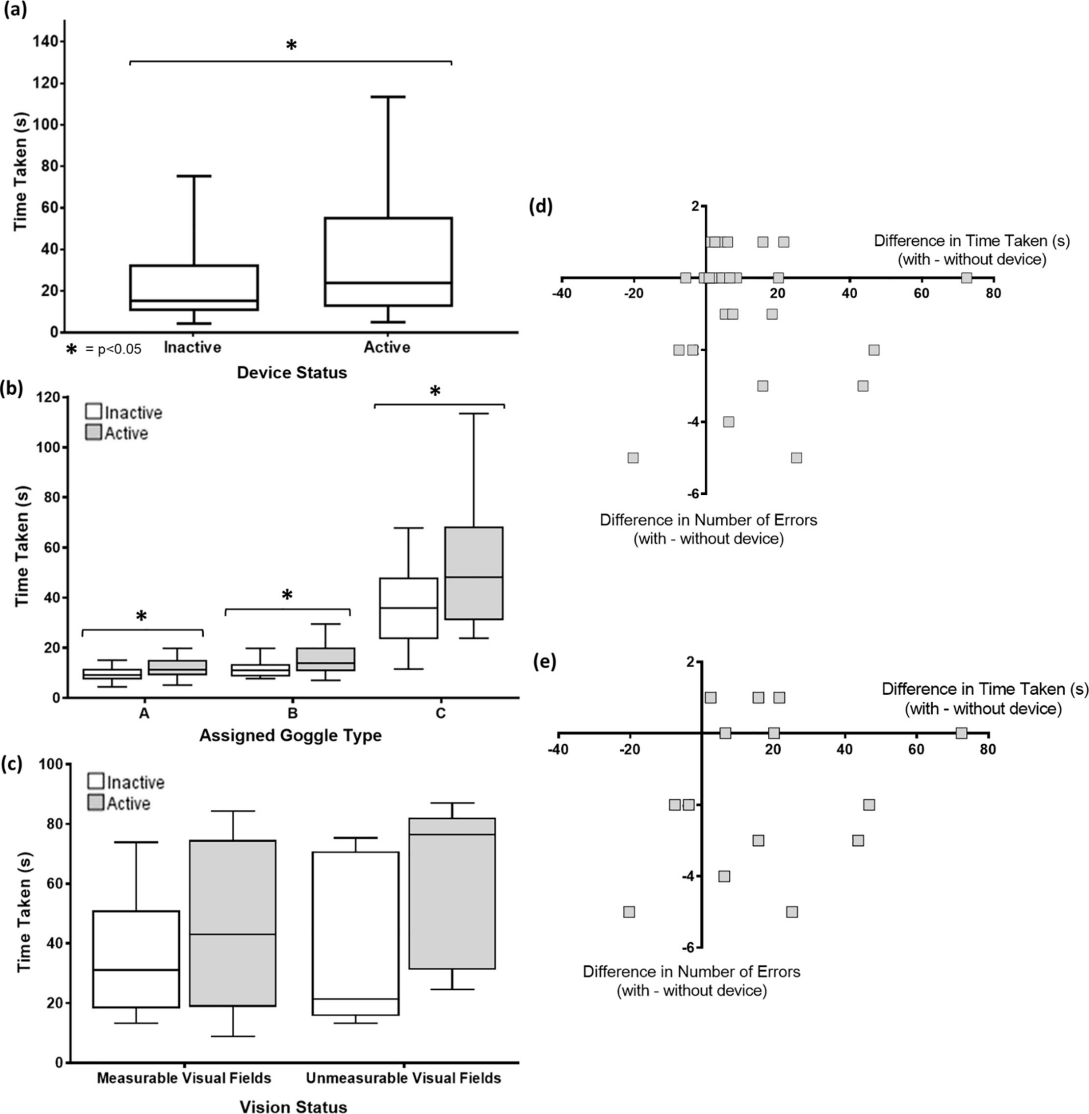
Time taken to complete mazes. Time taken to complete the mazes increased when using the LEO Belt compared to without the LEO Belt. This occurred for all subjects regardless of the stage of reduced peripheral vision (p<0.001) (a). Sighted subjects were slower when using the device (grey) whilst wearing goggle A (p = 0.002), goggle B (p = 0.017) and goggle C (p = 0.018) compared to without (white) (b). The mean increase in time taken was greater for visually impaired subjects with unmeasurable VFs compared to measurable VFs (c). Time taken was not significantly correlated with number of errors made by sighted subjects (p = 0.694) (d). This was also true when focusing only on those wearing goggle C (p = 0.47) (e).

Time taken to complete the mazes increased when using the LEO Belt for all subjects by an average of 10.7 seconds (p<0.001) (Fig 5A). Separating sighted subjects by goggle, time taken was significantly increased for goggles A, B and C by 24, 38 and 47% respectively (Fig 5B). Maze data from visually impaired subjects was analysed by studying trends as the small sample size meant statistical calculations could not be performed. Visually impaired subjects were also slower when using the device, most notably in those with unmeasurable VFs (Fig 5C). Change in time taken was not significantly correlated to change in number of errors made with and without the LEO Belt for all sighted subjects (p = 0.694) (Fig 5D) and only those wearing goggle C (p = 0.47) (Fig 5E).

**Fig 6:**
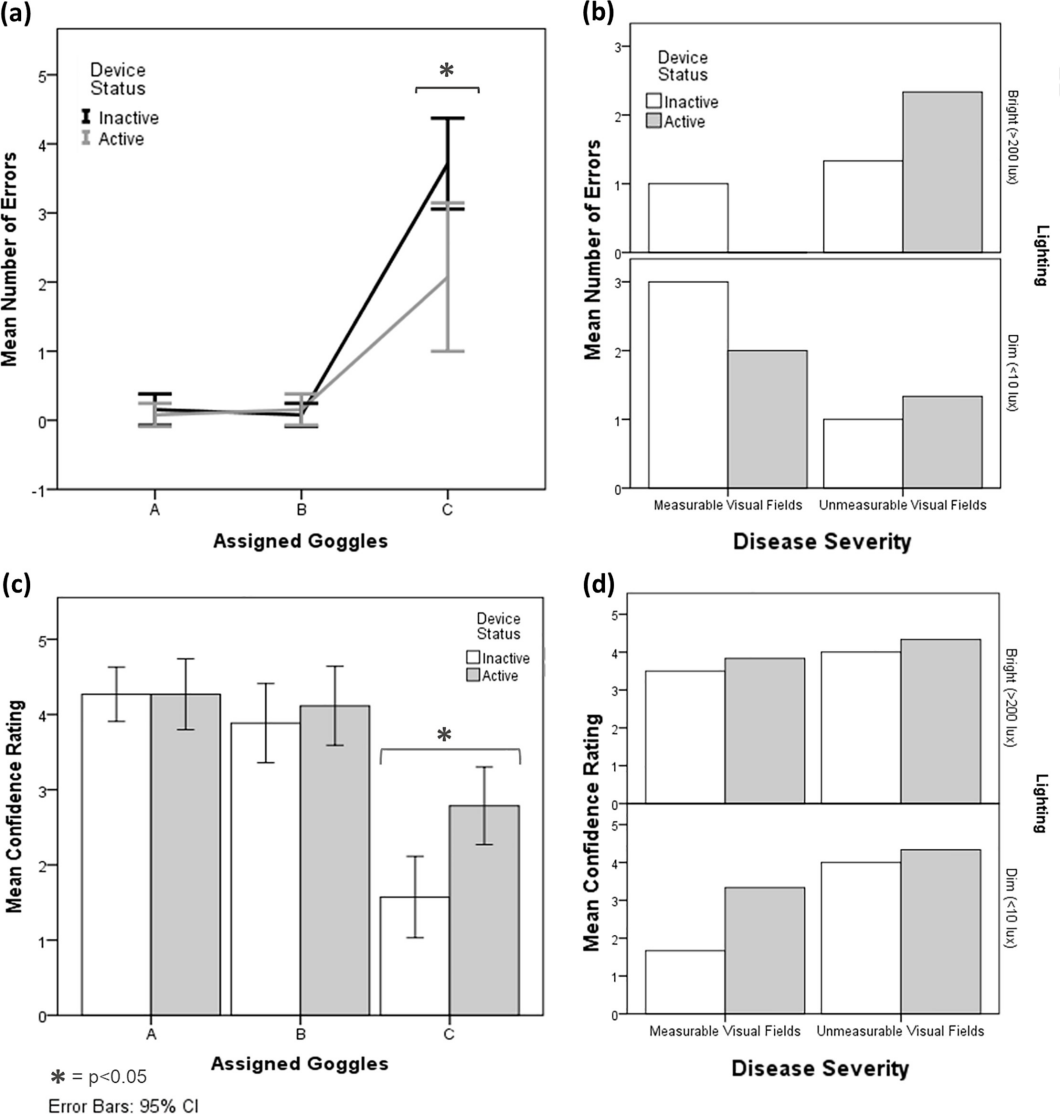
Change in number of errors and confidence scores when using the LEO Belt. Results from sighted subjects show significant decrease in number of errors when wearing goggle C (p = 0.014) with the LEO Belt (grey) compared to without it (white) (a). Trends from visually impaired subjects show reduced errors in the group with measureable VFs, but increased errors in those with unmeasurable VFs, when using the LEO Belt (b). Self-reported confidence of sighted subjects increased when using the device when wearing goggle C (p = 0.004) (c). Visually impaired subjects had increased confidence overall, most notably in the group with measureable VFs in dim lighting (d).

Sighted subjects experienced a negligible change in number of errors made when using the device whilst wearing goggle A or B (mean difference in number of errors = <0.1 and p = 0.564 for both), but errors reduced whilst wearing goggle C (mean reduction in errors = 1.6, p = 0.014) (Fig 6A). For visually impaired subjects with unmeasurable VFs, mean number of errors increased when using the device whilst those with measurable VFs had reduced mean errors from two to one. This group also made fewer errors in bright lighting overall; however, improvement when using the LEO Belt was equivalent in both bright and dim light (Fig 6B).

Subjects rated confidence on a scale from no confidence (= 1) to extremely confident (= 5) following each maze attempt. Confidence scores generally decreased with reducing VFs in the sighted subject group. The LEO Belt had no significant effect on confidence scores whilst wearing goggle A (0% increase in confidence, p = 1.00) or B (6% increase in confidence, p = 0.3), but caused increased confidence when wearing goggle C (77% increase in confidence, p = 0.004) (Fig 6C). For the visually impaired group, overall confidence increased when using the device by a mean of 0.7. This was particularly notable in the half with measurable VFs, whose confidence doubled when using the device in dim lighting. Those with unmeasurable VFs had higher reported confidence throughout compared to those with measurable VFs (Fig 6D).

**Fig 7:**
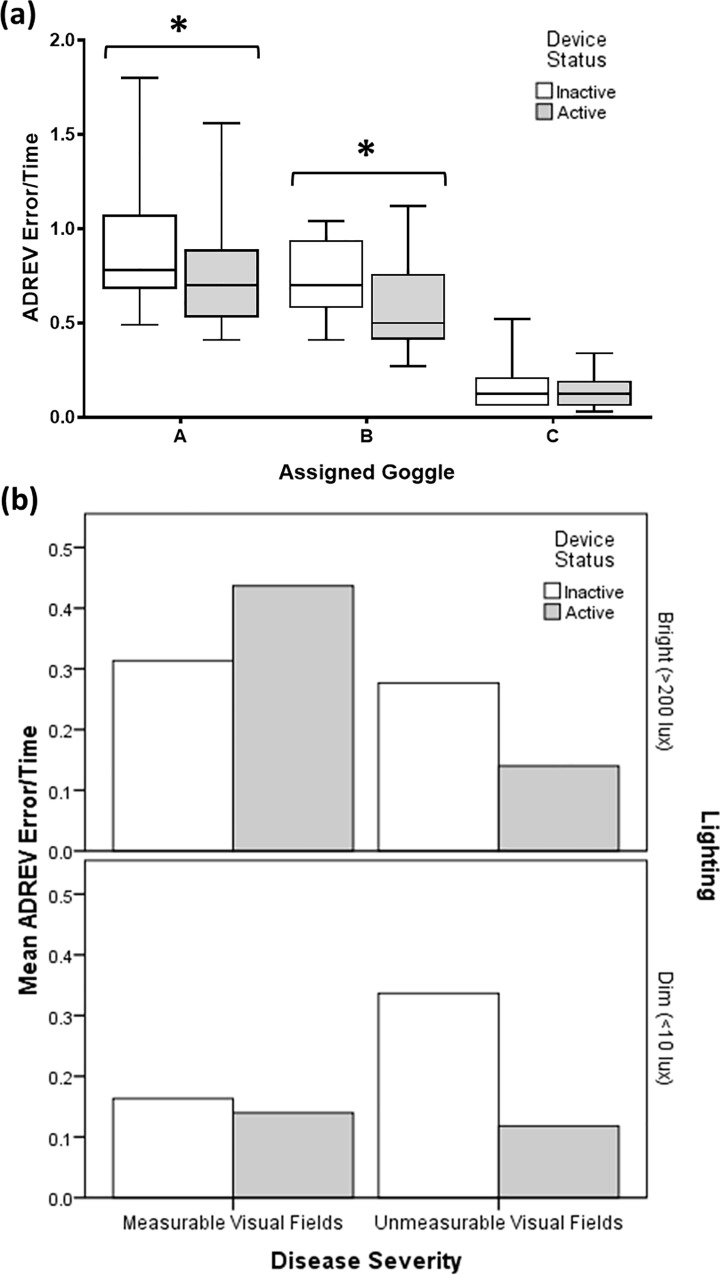
ADREV Error/Time results. Sighted subject results suggest poorer performance when using the device (grey) compared to not (white) whilst wearing goggle A (p = 0.001) and B (p = 0.005) but no change with goggle C (p = 0.944) (a). Visually impaired subjects with measurable VFs performed better with the device on average whilst those with unmeasurable VFs performed worse (b).

Combining results as ADREV error/time scores, sighted subjects wearing the LEO Belt had reduced scores, reflecting a poorer performance, whilst wearing goggle A (mean reduction = 0.16, p = 0.001) and B (mean reduction = 0.17, p = 0.005). There was no statistically significant change when wearing goggle C (mean difference = <0.01, p = 0.9). Visually impaired subjects with measurable VFs had improved ADREV error/time scores when using the device in bright lighting conditions. Those with unmeasurable VFs more than halved their ADREV error/time scores (Fig 7).

**Fig 8:**
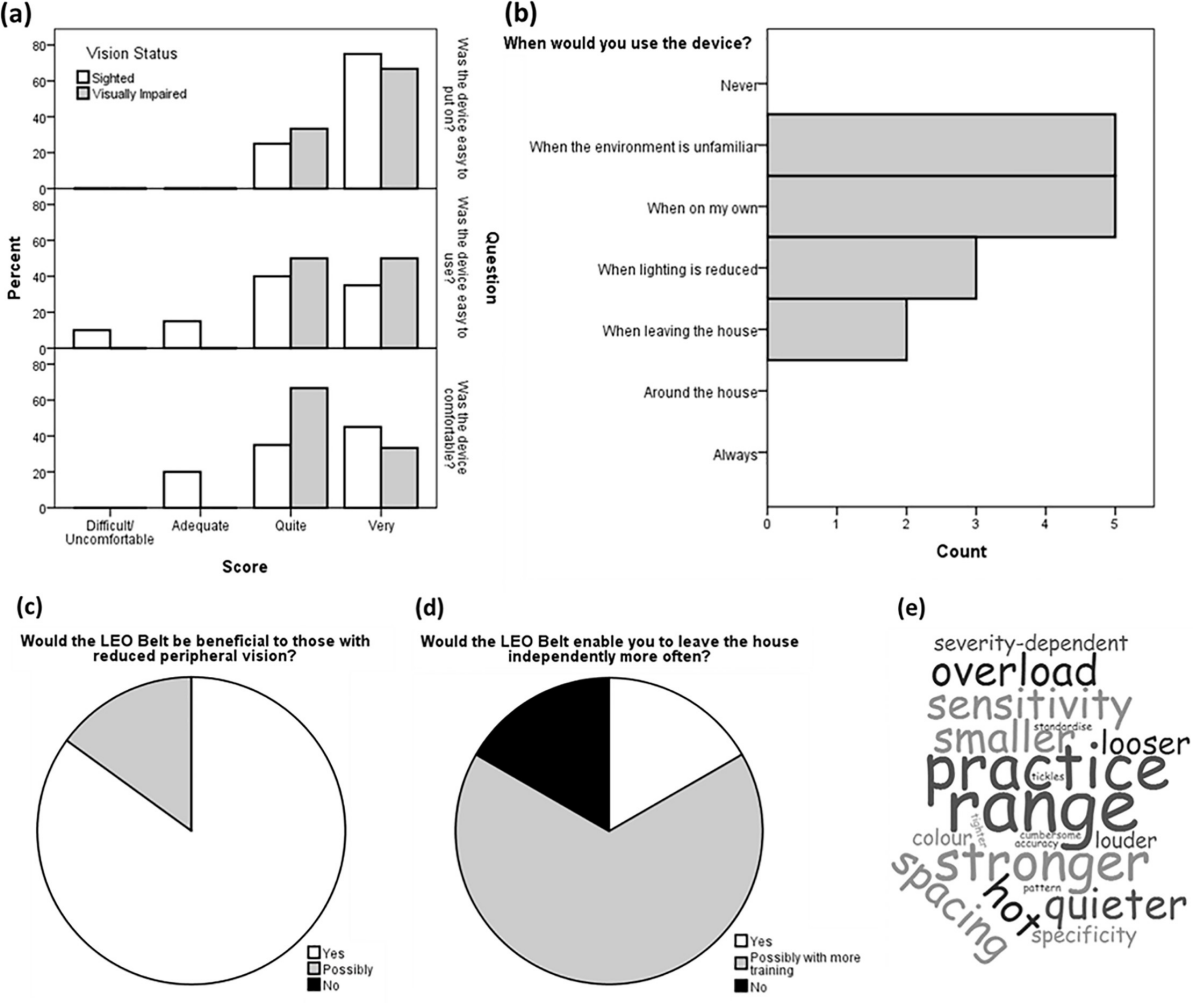
Questionnaire feedback. Questionnaire responses revealed user opinions and suggestions for improvements following using the LEO Belt. Opinions were collected from both sighted (white) and visually impaired (grey) subjects on ease to wear, use and comfort of the device (a). Visually impaired subjects were also asked if they owned the device when they would use it (b). Sighted and visually impaired subjects were both asked their views on the future usefulness of the device to others (c) or themselves (d) respectively. All subjects offered suggestions to improve the LEO Belt, shown in a word cloud (e).

All subjects were at least adequately satisfied with the ease of wear and comfort of the device (100%) and 92.3% found it relatively easy to use (Fig 8A). Visually impaired subjects identified the LEO Belt would be most useful when navigating unfamiliar environments and when alone (Fig 8B). 85% of sighted subjects thought the LEO Belt would be beneficial as a visual aid to those with reduced vision, and 15% were unsure (Fig 8C). The vast majority of visually impaired subjects (except one, S2 Fig) felt the device would increase their independence compared to their current aid (Fig 8D). The most frequent suggestion to improve the device was to alter the range; referring to the area the camera can detect (Fig 8E).

## Discussion

This study sought to determine whether the LEO Belt has the capacity to become a new visual aid for those with reduced peripheral vision. All subjects were significantly slower navigating the mazes when using the LEO Belt however; those with reduced peripheral vision with measurable VFs experienced reduced number of errors. Self-reported confidence improved overall when using the LEO Belt, particularly for those with measurable VFs in dim lighting.

The slower navigation when using the LEO Belt contrasts to results from Pundlik’s collision warning device, also designed for those with reduced peripheral vision, which functions by beeping upon imminent collision.[8] In their experiment, speed to complete the maze did not significantly change when using the device.[8] An explanation for this difference is that haptic information may be more difficult to interpret than auditory information.[32] However, the LEO Belt’s haptic nature is especially advantageous for those with syndromic hearing loss such as Usher syndrome, present in 14% of RP patients [15] and half of this visually impaired cohort. Long-term tactile training has been proven to result in activation of the primary visual cortex upon stimulation, suggesting neuroplasticity.[33] Therefore, navigation speed when using the LEO Belt may increase with practice. Furthermore, slower speeds of navigation did not cause the improvement in number of errors when using the LEO Belt. Even upon isolating the group with significant error improvement (Fig 5E), reduced speed was not correlated to reduced number of errors.

Based upon the reduced number of errors in sighted subjects wearing goggle C (Fig 6A), it was anticipated that visually impaired subjects with the worst vision (those with unmeasurable VFs) would benefit most from using the device. However, number of errors actually increased for this group and reduced for those with measurable VFs (Fig 6B). At face value, the measurable VF cohort might be considered equivalent to goggle B used for sighted subjects, however, this cohort actually represents an intermediate impairment between goggle B and C. Due to their visual disease, those with measurable VFs not only had severe reduced peripheral vision, like goggle B, but also poor VA. This factor could not be modelled in the sighted cohort. This reinforces the imperative to test subjects with visual disease versus prior studies which used blindfolded-sighted individuals.[34–36] The LEO Belt device delivers simple directional and distance information, hence subjects with measurable VFs benefit most as their remaining central vision can be directed towards objects they otherwise would miss.

The increase in confidence found in dim lighting for the visually impaired cohort with measurable VFs (Fig 6D) correlated to self-reported nyctalopia (Table 1). Questionnaire data also suggests the LEO Belt may moderate the effects of nyctalopia on activity, as half the visually impaired subjects stated they would use the device when lighting is reduced (Fig 8B). Visually impaired subjects with the worst vision were more confident overall (Fig 6D), possibly due to familiarity with relying completely on a visual aid. One weakness of using this subject-reported outcome is that there is subjectivity in reporting confidence, especially as testing occurred in a controlled situation with a researcher observing. Testing at home trials therefore may be more reflective of true confidence. Another limitation of the study is some subjects used the LEO Belt as a visual aid in isolation and others used it as an adjunct. It has potential to be beneficial as both but further studies with larger number of subjects should determine its most suitable use.

This study is one of the first to include user opinions when determining the success of an ETA. This approach is important to try to address the issue that ETAs tested in research settings are not being translated into everyday use.[5, 21] Previous literature introducing new devices largely do not include any testing on subjects [37–39] or focus on mobility data without considering subject feedback.[8, 34, 40] This study recruited subjects from a single tertiary centre and sample size of the visually impaired cohort was small. Further studies with larger cohorts would be required to validate results. Despite this, their opinions concerning wearability, usability and comfort followed the positive trends of sighted subjects (Fig 8A). The LEO Belt also has potential to increase independence, a widely reported consequence of reduced vision,[26] however the majority felt more training was required to achieve this (Fig 8D). The importance of practice was also recognised in suggestions for improvement (Fig 8E) and in previous discussion to increase speed. Further testing and refinement of the prototype is recommended at home and in outdoor settings to determine its’ full potential.

A mobility assessment tool was required and a few standardised tools exist, including ADREV and Multi-luminance Mobility Test (MLMT), with the latter utilising seven luminance levels to bracket the level of nyctalopia.[41] Both mobility tests include high numbers of obstacles, 21 and 15 respectively, hence requiring specific equipment.[19, 41] Aspects of both were incorporated to create a simpler tool that was easy to set up, reproduce and specific for determining benefits to RP patients. It applied the scoring system from ADREV and altered lighting as proposed by MLMT. The lux values used to represent bright light, >200 lux, and dim light, <10 lux, were chosen to reflect illuminance levels of a well-lit room and dusk, respectively.[41] As the sighted group have no rod-photoreceptor pathology, testing altered lighting would have been futile. Instead, each sighted subject sequentially wore two of the three pairs of goggles so that all subjects undertook equal numbers of maze attempts. Some subjects with unmeasurable VFs had such poor vision as to no longer experience effects of nyctalopia (Table 1); arguably rendering this methodology unsuitable for them. Furthermore, the mazes only included limited types of obstacles. However, floor-based objects were prioritised as they are the most likely cause of falls, which have been shown to reduce independence and QOL.[42]

ADREV error/time scores were included as previous literature found it to be the most reliable predictor of visual disability.[20] In this study, these scores gave some indication regarding which severity of visual impairment benefited most from using the LEO Belt, however, combining error and time meant improvement in one or the other was obscured. Therefore, in determining whether an intervention can moderate the extent of visual disability, the usefulness of ADREV error/time scores is limited.

This study only included sight-limited subjects with RP, enabling easier comparison to sighted subjects as the goggles used to reduce their vision were modelled on RP disease topography and progression. Furthermore, analysis was simpler as it reduced the number of differences between visually impaired subjects. However, the LEO Belt’s usefulness may not be confined to those with RP and further testing of additional cohorts of diseased patients with restricted or impaired peripheral vision will be needed to determine generalisability.

## Conclusions

This pilot study has successfully determined the target group of the LEO Belt; those with severe reduced peripheral vision but still some functional central vision. These subjects experienced improved accuracy, and therefore confidence, when navigating. The device also shows potential to improve confidence in dim light for those reporting nyctalopia and in increasing independence generally. As the first study of a wearable, tactile visual aid tested in a patient cohort, the LEO Belt proves this concept is feasible and has high user acceptability.

## Supporting information

S1 Table Analysis of input sensors for ETAs. Summary of the benefits and limitations of the types of input sensors currently used for ETAs, adapted from Nguyen.[27].
(TIF)

S2 Table Artificially reducing the vision of sighted subjects. Three pairs of goggles were used to artificially reduce the vision of sighted subjects. The googles represented early (A), intermediate (B) and late (C) stages of RP disease progression. An Octopus perimeter machine was used whilst wearing the goggles to produce the visual field results.
(TIF)

S3 Table Visual field results. Visual field results for visually impaired subjects V04, V05 and V06. Goldmann visual field perimetry is the preferred method to clinically assess visual fields in patients with low vision or complex scotomas.[43] Subjects with very low VA, (V01, V02 and V03) were unable to see more than hand movements and hence were unable to have their visual fields measured in the last five years. Despite this, each subject had a reported history of reducing peripheral vision and a clinical diagnosis of RP.
(TIF)

S1 Fig Questionnaire form. Section 1 was completed following the first two maze attempts and Section 2 was completed at the end. Sighted subjects received a shorter version of this questionnaire, with non-applicable questions removed. When required, visually impaired subjects completed the questionnaire verbally.
(TIF)

S2 Fig Feedback from visually impaired subjects. Opinions and comments expressed during completion of the questionnaire following testing.
(TIF)

## References

[1] Liew G, Michaelides M, Bunce C. A comparison of the causes of blindness certifications in England and Wales in working age adults (16–64 years), 1999–2000 with 2009–2010. BMJ Open. 2014;4(e004015). 10.1136/bmjopen-2013-004015 24525390 PMC3927710

[2] Russell S, Bennett J, Wellman JA, Chung DC, Yu Z-F, Tillman A, et al Efficacy and safety of voretigene neparvovec (AAV2-hRPE65v2) in patients with RPE65-mediated inherited retinal dystrophy: a randomised, controlled, open-label, phase 3 trial. The Lancet. 2017;390(10097):849–60. 10.1016/S0140-6736(17)31868-8. PMC5726391 28712537

[3] U.S. Food and Drug Administration (FDA). FDA approves novel gene therapy to treat patients with a rare form of inherited vision loss [WebContent]. 2017 [28th August 2018]. Available from: https://www.fda.gov/newsevents/newsroom/pressannouncements/ucm589467.htm.

[4] Elmannai W, Elleithy K. Sensor-based assistive devices for visually-impaired people: current status, challenges, and future directions. Sensors (Basel). 2017;17(3). Epub 2017/03/14. 10.3390/s17030565 28287451 PMC5375851

[5] Kim Y, Harders M, Gassert R. Identification of Vibrotactile Patterns Encoding Obstacle Distance Information. IEEE Transactions on Haptics. 2015;8(3). 10.1109/TOH.2015.2415213 25807569

[6] Intel Corporation. Intel RealSense Spatial Awareness Wearable (IRSAW) GitHub2015 [27th August 2018]. Available from: https://github.com/IRSAW/IRSAW.

[7] Kaplan K. Technology Brings Spatial Awareness to People with Vision Loss: iQ by Intel; 2017 [updated 2017-02-17; cited 2017 24th November 2017]. Available from: https://iq.intel.com/technology-brings-spatial-awareness-to-the-visually-impaired/.

[8] Pundlik S, Tomasi M, Luo G. Evaluation of a Portable Collision Warning Device for Patients With Peripheral Vision Loss in an Obstacle Course. Investigative Ophthalmology and Visual Science. 2015;56(4):2571–9. Epub 2015/03/20. 10.1167/iovs.14-15935 . 25788655

[9] Haddrill M, Heiting G. Peripheral Vision Loss: Tunnel Vision Causes and Treatments AllAboutVision.com: @AllAboutVision; 2017 [cited 2017 11/11/2017]. Available from: http://www.allaboutvision.com/conditions/peripheral-vision.htm.

[10] Nation Eye Institute. Facts About Retinitis Pigmentosa: National Eye Institute; 2018 [3rd July 2018]. Available from: https://www.ncbi.nlm.nih.gov/pubmed/.

[11] Grover S, Fishman GA, Brown J. Patterns of visual field progression in patients with retinitis pigmentosa. Ophthalmology. 1998;105(6):1069–75. 10.1016/S0161-6420(98)96009-2 9627658

[12] Herse P. Retinitis pigmentosa: visual function and multidisciplinary management. Clinical and Experimental Optometry. 2005;88(5):335–50. Epub 2005/11/01. . 16255692 10.1111/j.1444-0938.2005.tb06717.x

[13] Fahim AT, Daiger SP, Weleber RG. Nonsyndromic Retinitis Pigmentosa Overview In: Adam M, Ardinger H, Pagon R, editors. Gene Reviews^®^ [Internet]. Seattle: University of Washington, Seattle; 2000 [updated 2017]. 20301590

[14] Ferrari S, Di Iorio E, Barbaro V, Ponzin D, Sorrentino FS, Parmeggiani F. Retinitis Pigmentosa: Genes and Disease Mechanisms. Current Genomics. 122011. p. 238–49. 10.2174/138920211795860107 PMC3131731 22131869

[15] Hamel C. Retinitis pigmentosa. Orphanet Journal of Rare Diseases. 2006;1:40 Epub 2006/10/13. 10.1186/1750-1172-1-40 17032466 PMC1621055

[16] Yanagisawa M, Kato S, Kobayashi M, Watanabe M, Ochiai M. Relationship between vision-related quality of life and different types of existing visual fields in Japanese patients. International Ophthalmology. 2012;32(6):523–9. Epub 2012/05/15. 10.1007/s10792-012-9581-x . 22581307

[17] Latham K, Baranian M, Timmis MA, Fisher A, Pardhan S. Relative Difficulties of Daily Living Tasks with Retinitis Pigmentosa. Optometry and Vision Science. 2017;94(3):317–28. 10.1097/OPX.0000000000001046 28033161

[18] Black A, Lovie‐Kitchin JE, Woods RL, Arnold N, Byrnes J, Murrish J. Mobility performance with retinitis pigmentosa. Clinical and Experimental Optometry. 1997;80(1):1–12. 10.1111/j.1444-0938.1997.tb04841.x

[19] Lorenzana L, Lankaranian D, Dugar J, Mayer J, Palejwala N, Kulkarni K, et al A new method of assessing ability to perform activities of daily living: design, methods and baseline data. Ophthalmic Epidemiology. 2009;16(2):107–14. 10.1080/09286580902738142 19353399

[20] Warrian KJ, Katz LJ, Myers JS, Moster MR, Pro MJ, Wizov SS, et al A comparison of methods used to evaluate mobility performance in the visually impaired. British Journal of Ophthamology. 2015;99:113–8. 10.1136/bjophthalmol-2014-305324 25138757

[21] Maidenbaum S, Abboud S, Amedi A. Sensory substitution: closing the gap between basic research and widespread practical visual rehabilitation. Neuroscience and Biobehavioural Reviews. 2014;41:3–15. Epub 2013/11/28. 10.1016/j.neubiorev.2013.11.007 . 24275274

[22] Kristjánsson Á, Moldoveanu A, Jóhannesson Ó I, Balan O, Spagnol S, Valgeirsdóttir VV, et al Designing sensory-substitution devices: Principles, pitfalls and potential. Restorative Neurology and Neuroscience. 2016;34(5):769–87. 10.3233/RNN-160647 . 27567755 PMC5044782

[23] Whitmarsh LE. The Benefits of Guide Dog Ownership. Visual Impairment Research. 2005;7(1):27–42. 10.1080/13882350590956439

[24] National Federation of the Blind. Blindness Statistics 2017 [17th November 2017]. Available from: https://nfb.org/blindness-statistics.

[25] Maidenbaum S, Hanassy S, Abboud S, Buchs G, Chebat DR, Levy-Tzedek S, et al The "EyeCane", a new electronic travel aid for the blind: Technology, behavior & swift learning. Restorative Neurology and Neuroscience. 2014;32(6):813–24. Epub 2014/09/10. 10.3233/RNN-130351 . 25201814

[26] Pavey S, Dodgson A, Douglas G, Clements B. Travel, transport and mobility of people who are bind and partially sighted in the UK. University of Birmingham: Royal National Institute of Blind People (RNIB), 2009.

[27] Nguyen C. Haptic Obstacle Detector for the Blind. Stockholm: Karolinska Institutet; 2014.

[28] von Haller Gilmer B. Problems in Cutaneous Communication from Psychophysics to Information Processing. New York: American Foundation for the Blind; 1966. 40 p.

[29] Spence C. The skin as a medium for sensory substitution. Multisensory Research. 2014;27(5–6):293–312. Epub 2015/02/20. . 25693298 10.1163/22134808-00002452

[30] Bhatlawande S, Sunkari A, Mahadevappa M, Mukhopadhyay J, Biswas M, Das D, et al Electronic bracelet and vision-enabled waist-belt for mobility of visually impaired people. Assistive Technology. 2014;26(4):186–95. Epub 2014/01/01. 10.1080/10400435.2014.915896 . 25771603

[31] Bach-y-Rita P, Kaczmarek KA, Tyler ME, Garcia-Lara J. Form perception with a 49-point electrotactile stimulus array on the tongue: a technical note. Journal of Rehabilitation Research and Development. 1998;35(4):427–30. Epub 1999/04/29. . 10220221

[32] Adebiyi A, Sorrentino P, Bohlool S, Zhang C, Arditti M, Goodrich G, et al Assessment of feedback modalities for wearable visual aids in blind mobility. PLoS One. 2017;12(2). 10.1371/journal.pone.0170531 28182731 PMC5300186

[33] Saito DN, Okada T, Honda M, Yonekura Y, Sadato N. Practice makes perfect: the neural substrates of tactile discrimination by Mah-Jong experts include the primary visual cortex. BMC Neuroscience. 2006;7:79 10.1186/1471-2202-7-79 . 17144928 PMC1698492

[34] Khampachua C, Wongrajit C, Waranusast R, Pattanathaburt P. Wrist-mounted smartphone-based navigation device for visually impaired people using ultrasonic sensing—IEEE Conference Publication. 2016 Fifth ICT International Student Project Conference (ICT-ISPC); Thailand: IEEE; 2016.

[35] Shoval S, Borenstein J, Koren Y. The NavBelt—a computerized travel aid for the blind based on mobile robotics technology. IEEE Trans Biomed Eng. 1998;45(11):1376–86. Epub 1998/11/07. 10.1109/10.725334 . 9805836

[36] Cardin S, Thalmann D, Vexo F. A wearable system for mobility improvement of visually impaired people. The Visual Computer. 2007;23(2):109–18. 10.1007/s00371-006-0032-4

[37] Johnson LA, Higgins CM. A Navigation Aid for the Blind Using Tactile-Visual Sensory Substitution. Engineering in Medicine and Biology Society (EMBS) Annual International Conference of the IEEE; New York, USA: IEEE; 2006. 10.1109/IEMBS.2006.259473 17945950

[38] Bahadir SK, Koncar V, Kalaoglu F. Wearable obstacle detection system fully integrated to textile structures for visually impaired people. Sensors and Actuators A: Physical. 2012;179(Supplement C):297–311. 10.1016/j.sna.2012.02.027.

[39] Dakopoulos D, Boddhu SK, Bourbakis NG. A 2D Vibration Array as an Assistive Device for Visually Impaired. Bioinformatics and Bioengineering IEEE International Conference; Boston, USA: IEEE; 2007.

[40] Chebat DR, Maidenbaum S, Amedi A. Navigation using sensory substitution in real and virtual mazes. PLoS One. 2015;10(6):e0126307 Epub 2015/06/04. 10.1371/journal.pone.0126307 PMC4454637 26039580

[41] Chung DC, McCague S, Yu ZF, Thill S, DiStefano‐Pappas J, Bennett J, et al Novel mobility test to assess functional vision in patients with inherited retinal dystrophies. Clinical and Experimental Ophthalmology. 2017 10.1111/ceo.13022 28697537 PMC5764825

[42] Dhital A, Pey T, Stanford MR. Visual loss and falls: a review. Eye. 2010;24(9):1437–46. Epub 2010/05/08. 10.1038/eye.2010.60 . 20448666

[43] Dersu I, Wiggins MN, Luther A, Harper R, Chacko J. Understanding Visual Fields, Part I: Goldmann Perimetry. Journal of Ophthalmic Medical Technology. 2006;2(2).

