# Supplementary figures and images for: A novel, wearable, electronic visual aid to assist those with reduced peripheral vision
Source: PLoS One. 2019 Oct 15;14(10):e0223755. doi: 10.1371/journal.pone.0223755 (PMC6793879; doi:10.1371/journal.pone.0223755)

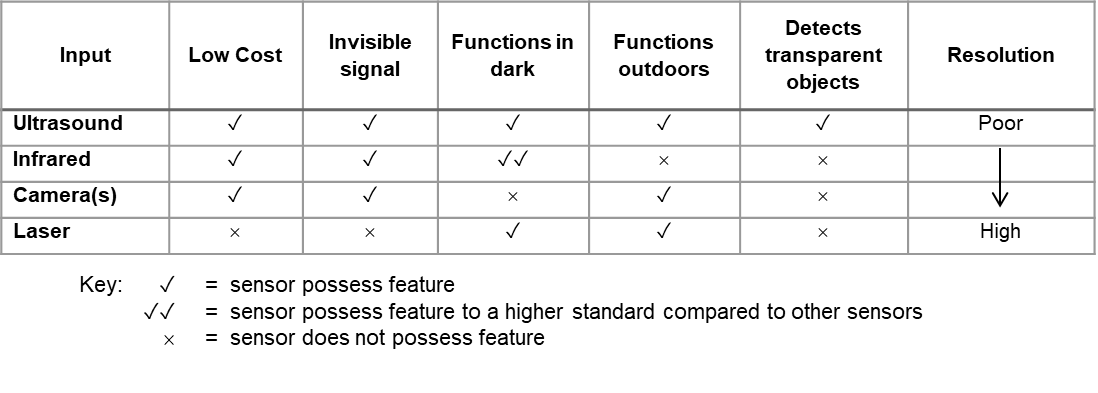

Supplement: S1 Table — Summary of the benefits and limitations of the types of input sensors currently used for ETAs, adapted from Nguyen.[27]. (TIF) [file pone.0223755.s001.tif]

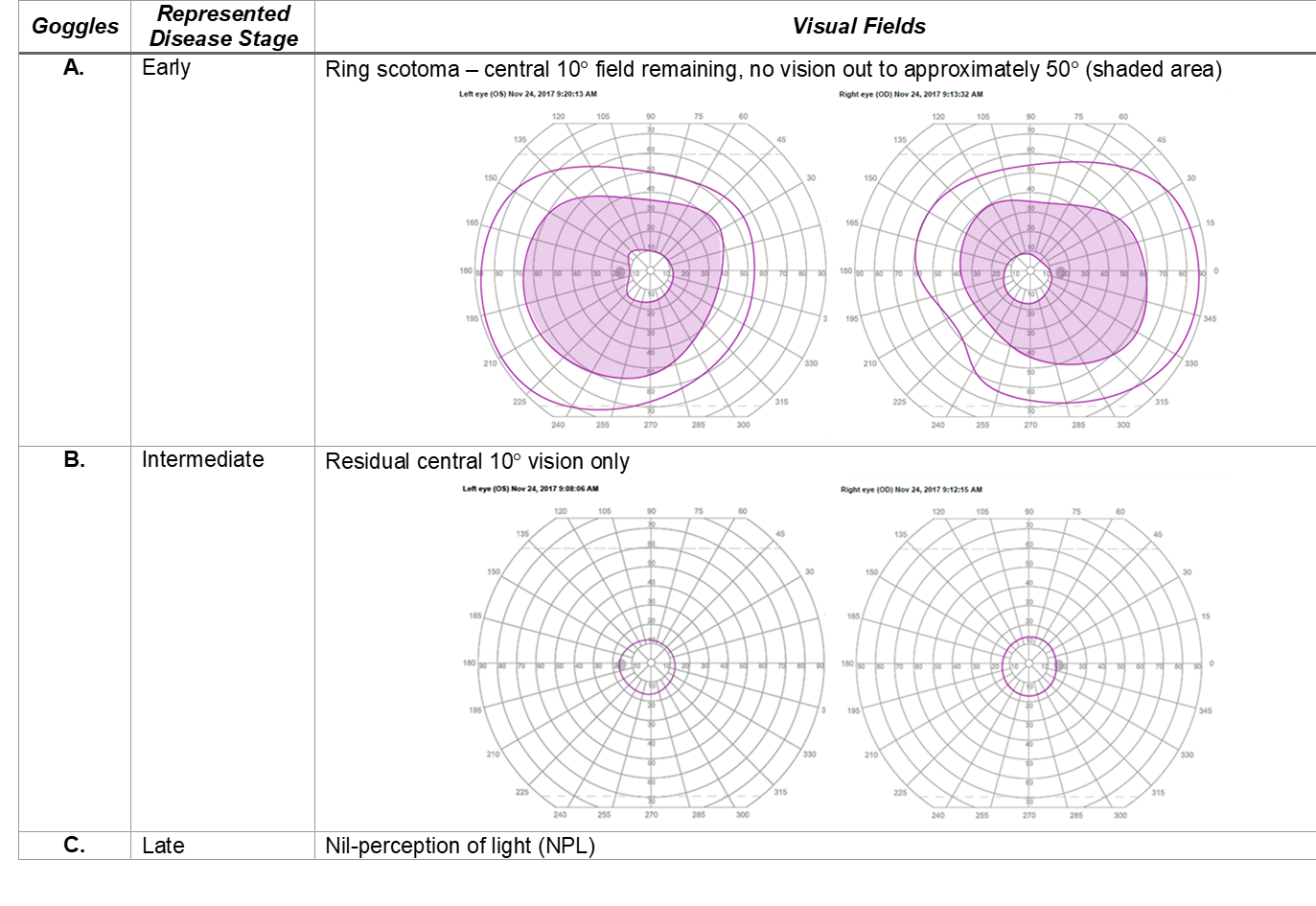

Supplement: S2 Table — Three pairs of goggles were used to artificially reduce the vision of sighted subjects. The googles represented early (A), intermediate (B) and late (C) stages of RP disease progression. An Octopus perimeter machine was used whilst wearing the goggles to produce the visual field results. (TIF) [file pone.0223755.s002.tif]

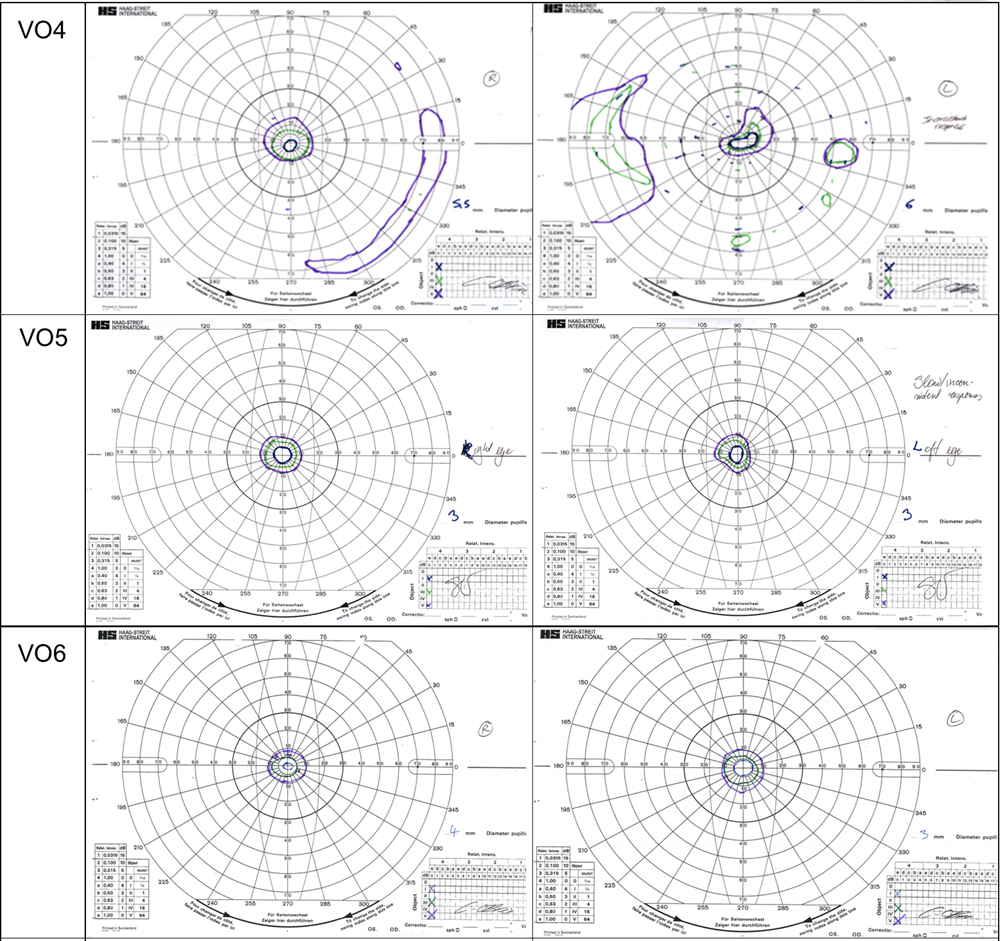

Supplement: S3 Table — Visual field results for visually impaired subjects V04, V05 and V06. Goldmann visual field perimetry is the preferred method to clinically assess visual fields in patients with low vision or complex scotomas.[43] Subjects with very low VA, (V01, V02 and V03) were unable to see more than hand movements and hence were unable to have their visual fields measured in the last five years. Despite this, each subject had a reported history of reducing peripheral vision and a clinical diagnosis of RP. (TIF) [file pone.0223755.s003.tif]

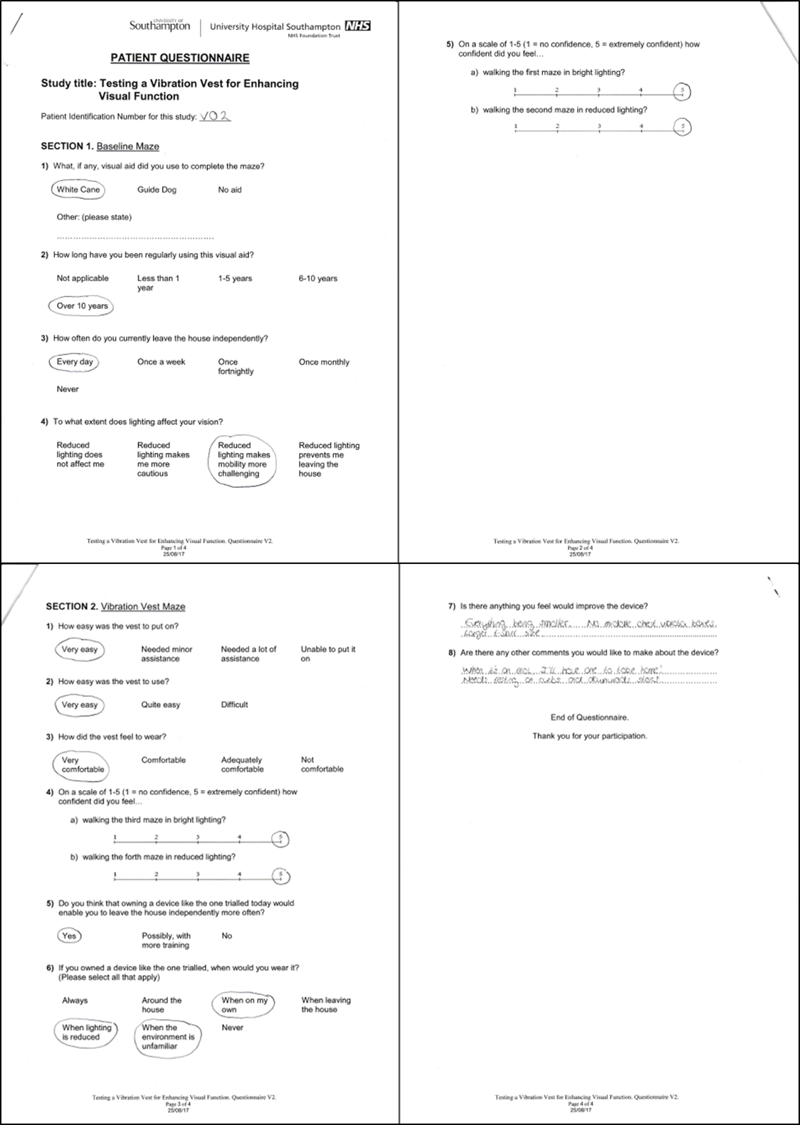

Supplement: S1 Fig — Section 1 was completed following the first two maze attempts and Section 2 was completed at the end. Sighted subjects received a shorter version of this questionnaire, with non-applicable questions removed. When required, visually impaired subjects completed the questionnaire verbally. (TIF) [file pone.0223755.s004.tif]

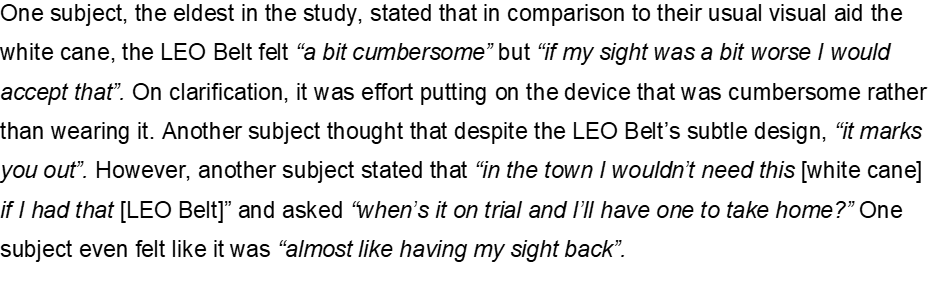

Supplement: S2 Fig — Opinions and comments expressed during completion of the questionnaire following testing. (TIF) [file pone.0223755.s005.tif]
